# Retinal vessel architecture and geometry are not impaired in normal-tension glaucoma

**DOI:** 10.1038/s41598-023-33361-2

**Published:** 2023-04-25

**Authors:** Anne-Sophie Leveque, Magali Bouisse, José Labarere, Emanuele Trucco, Stephen Hogg, Tom MacGillivray, Florent Aptel, Christophe Chiquet

**Affiliations:** 1 Department of Ophthalmology, University Hospital of Grenoble, Grenoble Alpes University Hospital, CS 10217, 38043 Grenoble Cedex 09, France; 2 Clinical Epidemiology Unit, Grenoble Alpes University Hospital, Grenoble, France; 3 Univ. Grenoble Alpes, CNRS, UMR 5525, TIMC, Grenoble, France; 4 VAMPIRE Project, Computing, School of Science and Engineering, University of Dundee, Dundee, UK; 5 VAMPIRE Project, Centre for Clinical Brain Science, University of Edinburgh, Edinburgh, UK; 6 Univ. Grenoble Alpes, HP2 Laboratory, INSERM U1300, Grenoble, France

**Keywords:** Translational research, Optic nerve diseases

## Abstract

To investigate the associations between retinal vessel parameters and normal-tension glaucoma (NTG). We conducted a case–control study with a prospective cohort, allowing to record 23 cases of NTG. We matched NTG patient with one primary open-angle glaucoma (POAG) and one control per case by age, systemic hypertension, diabetes, and refraction. Central retinal artery equivalent (CRAE), central retinal venule equivalent (CRVE), Arteriole-To-Venule ratio (AVR), Fractal Dimension and tortuosity of the vascular network were measured using VAMPIRE software. Our sample consisted of 23 NTG, 23 POAG, and 23 control individuals, with a median age of 65 years (25–75th percentile, 56–74). No significant differences were observed in median values for CRAE (130.6 µm (25–75th percentile, 122.8; 137.0) for NTG, 128.4 µm (124.0; 132.9) for POAG, and 135.3 µm (123.3; 144.8) for controls, *P* = .23), CRVE (172.1 µm (160.0; 188.3), 172.8 µm (163.3; 181.6), and 175.9 µm (167.6; 188.4), *P* = .43), AVR (0.76, 0.75, 0.74, *P* = .71), tortuosity and fractal parameters across study groups. Vascular morphological parameters were not significantly associated with retinal nerve fiber layer thickness or mean deviation for the NTG and POAG groups. Our results suggest that vascular dysregulation in NTG does not modify the architecture and geometry of the retinal vessel network.

## Introduction

Normal-tension glaucoma (NTG) is a chronic neuropathy characterized by progressive change in the optic nerve head and subsequent visual field defects. The pathophysiology of NTG is complex and multifactorial. Intraocular pressure (IOP) is within statistical limits of normal (10–21 mmHg) however the level of IOP is a risk factor for progression^1^. Other potential risk factors have been identified including familial history, ethnicity, females, and vascular dysregulation^2–4^.

Investigators have reported an association between NTG, migraine and low systolic blood pressure, suggesting a possible role of vascular components in the pathogenesis of NTG^5^. Low blood pressure values measured during follow-up is correlated with structural degradation in medically treated NTG eyes^6^. Blood flow abnormalities have been reported in NTG, at the anterior part of the optic nerve head (ONH)^7^, the retrobulbar part of the optic nerve (ON)^8,9^, the choroid^10^ and the retina^11,12^. Vascular risk factors have been also implicated in the pathogenesis and progression of POAG^13^, and are recognized to be predominant in NTG.

Fundus camera imaging is a non-invasive method for imaging the retina, often used for measuring parameters of the vascular retinal network. The vascular morphological phenotype (including tortuosity and fractal dimension) provides information on the architecture and geometry of the vessel network, which determines the efficiency of blood circulation. VAMPIRE (Vessel Assessment and Measurement Platform for Images of the Retina, Universities of Edinburgh and Dundee) is a suite of validated software tools enabling a quantitative analysis of the vascular morphometry^14^. VAMPIRE has been used in a plethora of previous studies, e.g., on lacunar stroke, cognition, dementia and cardiovascular diseases^15–18^, and recently to investigate the retinal vessel phenotype in ocular diseases, such as non-arteritic ischemic optic neuropathy and primary open-angle glaucoma (POAG)^19,20^.

The objective of this study was to compare retinal vascular parameters for patients with NTG versus POAG and controls after controlling for heterogeneity in the prevalence of comorbid conditions and glaucoma severity.

## Materials and methods

### Study design

We conducted a single-center nested case–control study within the IMAGEYE cohort at Grenoble-Alpes University Hospital. This study complies with the declaration of Helsinki guidelines for research involving human subjects. Informed consent was obtained from the subjects after explanation of the study. Data collection was performed under a regime of consented participation, including the option for patients of withdrawing their data at any moment. Data were collected after obtaining patient consent except in case of declining participation by the patient or their relatives if unable to decline in person (IMAGEYE cohort, non-interventional study implicating the human person). The Institutional review board (*Comité de Protection des Personnes*, Sud Est V, Grenoble, France) reviewed and approved the study protocol, as part of the ongoing prospective observational IMAGEYE cohort study. This paper complied with the Strengthening the Reporting *of Observational Studies in Epidemiology* (STROBE) statement^21^.

### Patients

All included patients were recruited in the department of ophthalmology of University Hospital of Grenoble, during the period January 2020-April 2021. Twenty-one NTG were prevalent cases followed in our department whereas two NTG patients were incident cases. All POAG patients were prevalent cases.

Exclusion criteria were as follows: pregnant or lactating women, aged < 18 years, adult under guardianship or unable to consent, patients with high myopia (> -6 diopters) and any ocular disease except glaucoma. Further, group-specific exclusion criteria are listed in section study groups.

### Sample size

From 82 eligible patients (during the period January 2020-April 2021), 23 NTG, 23 POAG and 23 controls subjects were included. We excluded a further 4 POAG, 2 NTG patients and 4 controls because of poor quality of the fundoscopic image or images were rejected by VAMPIRE. Three NTG patients were also excluded due to high myopia.

### Data collection

Medical history was collected on the basis of self-declaration, particularly cardiovascular risk factors, high blood pressure, diabetes, dyslipidemia and smoking. IOP-lowering medications were also reported. All participants underwent a complete ocular examination including visual acuity (VA, LogMAR), objective refraction (Tonoref 2™, NIDEK SA, Gamagori, Japan) and measurement of IOP (non-contact tonometry, TONOREF II, Nidek™, Gamagori, Japan). Anterior segment slit lamp examination, gonioscopy and fundus examination were performed to rule out other ocular diseases.

#### Study groups

##### Normal-tension glaucoma patients

The diagnosis of NTG used the following criteria: diurnal measurements of IOP ≤ 21 mmHg, characteristic optic nerve damage including cup-to-disc ratio > 0.5, rim thinning or notching; retinal nerve fiber layer (RNFL) and ganglion cell complex (GCC) defect; corresponding VF loss with abnormal results on the Glaucoma Hemifield Test and pattern standard deviation outside 95% of normal limits. All subjects underwent magnetic resonance imaging (MRI) of the brain and a carotid Doppler test to rule out secondary causes of optic nerve damage.

##### Primary open-angle glaucoma patients

POAG were matched 1:1 with NTG patients for age (within 5 years), refraction (for myopia: − 1 to − 3D; − 3 to − 6D with exclusion of high myopia > − 6D), systemic hypertension, diabetes, and mean deviation (MD) of the visual field (0 to − 6 dB; − 6 to − 12 dB; − 12 to − 18 dB). Inclusion criteria for the POAG subjects were a history of IOP > 21 mmHg, open-angle on gonioscopy, glaucomatous optic nerve damage, RNFL and GCC damage associated with corresponding visual field damages. Secondary glaucoma and eyes with an history of acute angle closure glaucoma were excluded.

##### Controls

Controls were matched 1:1:1 with NTG and POAG subjects for age (within 5 years), refraction (for myopia: − 1 to − 3D; − 3 to − 6D with exclusion of high myopia > − 6D), systemic hypertension and diabetes. Inclusion criteria for the control group were IOP ≤ 21 mmHg and normal ocular examination. Optical coherence tomography (OCT) examinations, including RNFL and GCC measurements, were normal in these patients.

##### Visual field and optical coherence tomography examinations

Patients with NTG and POAG underwent visual field and OCT examinations. This data could not be collected for controls as OCT is not part of the routine follow-up examination. Humphrey 24.2 sita-standard visual field parameters (mean deviation (MD), pattern standard deviation (PSD) and visual field index (VFI)) were recorded (Zeiss Meditec, Inc, Dublin, CA, USA). Visual fields with fixation losses lower than 20%, false-positive lower than 33% and false-negative lower than 33% were considered reliable and used for the analysis for all glaucoma patients.

OCT examinations parameters, including ganglion cell complex (GCC), peripapillary and sectorial RNFL thickness, were acquired using a Cirrus HD-OCT 5000 (Zeiss Meditec, Dublin, CA, USA).

##### Fundus image analysis

45-degree funduscopic color photographs of both eyes were acquired, centered between the optic nerve and the macula after pupil dilatation, using two different non-mydriatic cameras: a Canon® CR-2 (30–2, Tokyo, Japan) with a resolution of 5184 × 3456 pixels and a Canon® CR-2 AF (9–1, Kawasaki, Kanagawa, Japan) with a resolution of 6000 × 4000 pixels. Funduscopic photographs were analyzed with the semi-automatic VAMPIRE software (version 3.2.0) by a single operator (ASL), after sitting the VAMPIRE operator training module.

**Figure 1:**
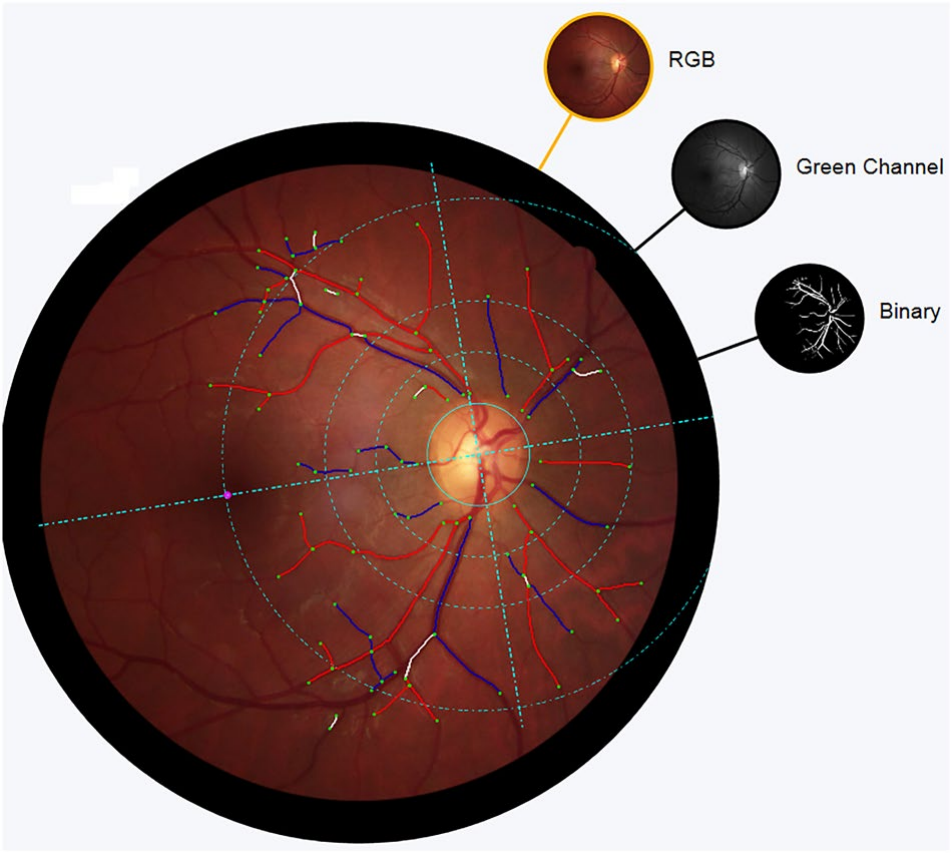
Example of retinal coordinates and zones used by Vampire. The definition of retinal coordinates (x axis through OD and macula centers, origin in the OD center) and circular concentric zones around the OD center, namely, zone A (between 0.5 and 1.0 optic disc diameter (ODD) from OD center), zone B (between 1.0 and 1.5 ODD from OD center) and zone C (between 1.0 and 2.5 ODD from OD center).

First, the optic disc (OD) and the fovea were located, with manual correction when necessary. This enabled the definition of retinal coordinates (x axis through OD and macula centers, origin in the OD center) and circular concentric zones around the OD center, namely, zone A (between 0.5 and 1.0 optic disc diameter (ODD) from OD center), zone B (between 1.0 and 1.5 ODD from OD center) and zone C (between 1.0 and 2.5 ODD from OD center, Fig. 1). Vessels were subsequently detected and labeled as arterioles or venules automatically and corrected manually where necessary.

CRAE and CRVE were computed as a weighted combinations of the widths of the six largest arteries and veins respectively using Knudtson’s revised formulas^22^ and arteriole-to venule ratio (AVR) as the ratio of CRAE to CRVE. The Fractal Dimension (FD) of the whole vascular network, a measure of the geometric complexity of the vessel network, was computed with the VAMPIRE implementation of a multifractal algorithm^23,24^. Arterial and venular vascular tortuosity was computed as the average of those of the six largest arteries and venules respectively) as defined elsewhere^25^. AVR, CRAE and CRVE were computed in zone B, fractal parameters and vascular tortuosity in zone C.

Raw measurements of CRAE and CRVE are computed in pixels by VAMPIRE. A pixel-to-microns conversion factor was obtained by dividing the average vertical ODD (over all images resized by VAMPIRE to a resolution of 3170 × 3170 pixels) by the assumed average of the disc diameter in microns (1850 μm), as previously described^22,26^. The conversion factor for the CANON CR-2 and CANON CR-2AF cameras was 3.9 microns per pixel.

##### Statistical analysis

Categorical data were reported as numbers and percentages and continuous variables summarized by median and 25th–75th percentiles. Comparisons across study groups were performed using the Kruskall-Wallis test for continuous variables and the Chi-square or Fisher exact test, as appropriate, for categorical variables. We used conditional logistic regression for paired samples to compare treatment categorical variables between NTG and POAG patients.

We used univariate linear regressions, modelling each retinal vessel parameter as a continuous dependent variable and RNFL and MD as continuous independent variables. We assessed the linearity assumption for continuous independent variables by using fractional polynomial functions. Two-sided p-values of < 0.05 were considered statistically significant. All analyses were performed using Stata Special Edition version 16.0 (Stata Corporation, College Station, TX, USA).

## Results

Of the 28 patients screened for eligibility, 23 cases of NTG were included. Our analytical sample consisted of 23 NTG patients, 23 POAG patients, and 23 controls matched by age (within 5 years), hypertension, diabetes mellitus, and refraction. The median age for all participants was 65 years (IQR, interquartile range (i.e., 25–75th percentiles): 56–74) and 40 (58%) were male (Table 1). Overall, the prevalence of hypertension, diabetes mellitus, current smoking, and dyslipidemia were 34.8%, 4.4%, 13.0%, and 27.5%, respectively. The median IOP was 12 mmHg (10–15).

**Table 1:** Comparison of baseline characteristics for Normal tension glaucoma (NTG), primary open-angle glaucoma (POAG) and control groups.

Characteristics	NTG		POAG		Control		*P*
	(n = 23)		(n = 23)		(n = 23)		
Age, median (IQR), years	63	(56; 74)	66	(59; 75)	65	(55; 74)	
Male, n (%)	16	(69.6)	15	(65.2)	9	(39.1)	0.08
Systemic hypertension, n (%)	8	(34.8)	8	(34.8)	8	(34.8)	
Diabetes, n (%)	1	(4.4)	1	(4.4)	1	(4.4)	
Refraction, median (IQR), diopters	+ 0.5	(− 1.75; + 1.25)	+ 0.0	(− 1.0; + 1.0)	+ 0.25	(− 1.5; + 1.5)	
Intraocular pressure, median (IQR), mmHg	11	(10; 15)	12	(10; 16)	13	(11; 15)	0.62
Smoking, n (%)	2	(8.7)	3	(13)	4	(17.4)	0.90
Dyslipidemia, n (%)	6	(26.1)	7	(30.4)	6	(26.1)	0.93
Pseudophakic, n (%)	4	(17.4)	5	21.7)	6	(26.1)	0.77
Topical medication, n (%)							
Beta blockers	11	(47.8)	15	(65.2)			0.18
Prostaglandins	19	(82.6)	20	(87.0)			0.71
Carbonic anhydrase inhibitors	3	(13)	12	(52.2)			< 0.01
Alpha 2 adrenergic agonists	2	(8.7)	4	(17.4)			0.42
SLT, n (%)	0	(0.0)	8	(34.8)			< 0.01
Filtering surgery, n (%)	0	(0.0)	2	(8.7)			0.16
MD, median (IQR), dB	− 5.1	(− 9.6; − 1.6)	− 6	(− 12.0; − 2.8)			
PSD, median (IQR), dB	5.6	(2.0; 10.4)	7.9	(5.2; 11.2)			0.046
RNFL, median (IQR), µm	73	(60; 78)	65	(54; 76)			0.37
GCC, median (IQR), µm	66	(55; 71)	61	(55; 71)			0.96

*GCC* Ganglion cell complex, *IQR* interquartile range (i.e., 25–75th percentiles), *MD* mean deviation, *PSD* pattern standard deviation, *RNFL* retinal nerve fiber layer, *SLT* selective laser trabeculoplasty.

Twenty NTG patients (87%) were treated with at least one hypotensive topical medication (Table 2). Twenty-one POAG patients (91%) were treated with at least one hypotensive topical medication (Table 2). NTG cases were less likely to receive therapy with carbonic anhydrase inhibitors and had lower median PSD values than their POAG counterparts. No other differences in demographics, cardiovascular risk factors, ocular examination, visual field and OCT parameters were found across study groups (Table 1).

**Table 2:** Comparison of treatment history between normal tension glaucoma (NTG) and primary open-angle glaucoma (POAG) groups.

	NTG *(n, %)*		POAG *(n, %)*	
Previous filtering surgery	0	(0%)	2	(8.70%)
Previous SLT	0	(0%)	8	(34.78%)
No treatment	3	(13.04%)	2	(8.70%)
Mono-therapy	10	(43.47%)	3	(13.04%)
Bi-therapy	6	(26.08%)	10	(43.47%)
Tri-therapy	3	(13.04%)	4	(17.39%)
Quadri-therapy	1	(4.35%)	4	(17.39%)

Glaucoma hypotensive medications included prostaglandins, beta blockers, carbonic anhydrase inhibitors and/or alpha 2 adrenergic agonists.

*SLT* selective laser trabeculoplasty.

**Figure 2:**
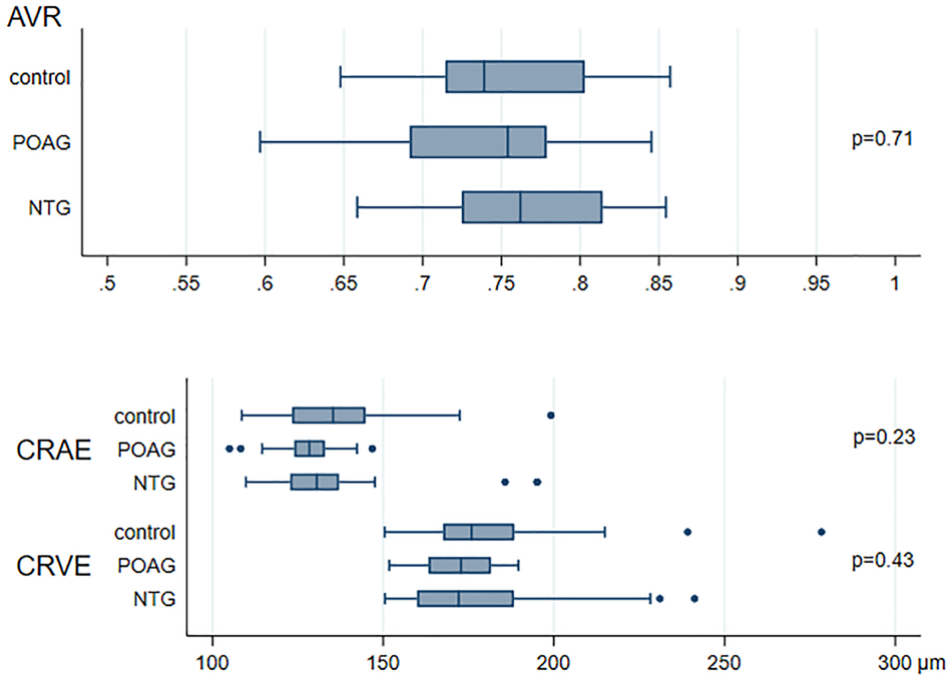
Bar graph of AVR, CRAE and CRVE values in the NTG, POAG and control groups. Data are expressed as median (interquartile range, 25–75th percentiles). *AVR* arteriole-to-venule ratio, *CRAE* central retinal arterial equivalent, *CRVE* central retinal venular equivalent, *NTG* normal tension glaucoma, *POAG* primary open angle glaucoma.

**Figure 3:**
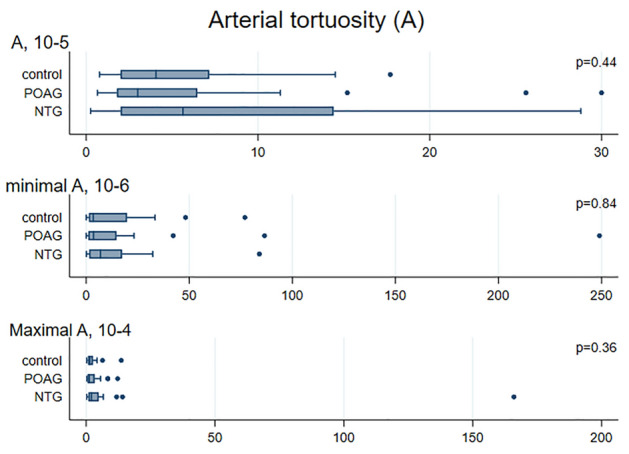
Bar graph of arterial tortuosity values in the NTG, POAG and control groups. Data are expressed as median (interquartile range, 25–75th percentiles). *NTG* normal tension glaucoma, *POAG* primary open angle glaucoma.

**Figure 4:**
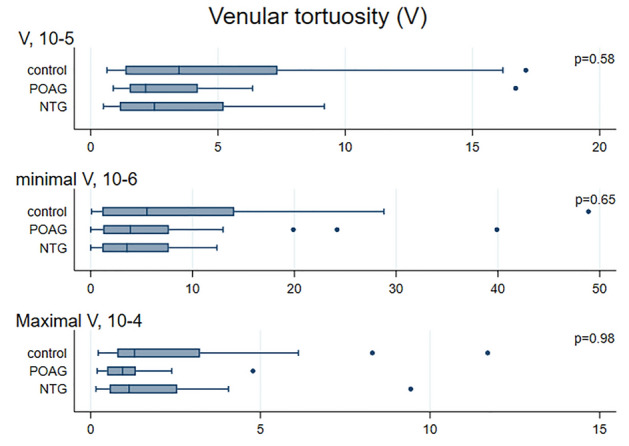
Bar graph of venular tortuosity values in the NTG, POAG and control groups. Data are expressed as median (interquartile range, 25–75th percentiles). *NTG* normal tension glaucoma, *POAG* primary open angle glaucoma.

**Figure 5:**
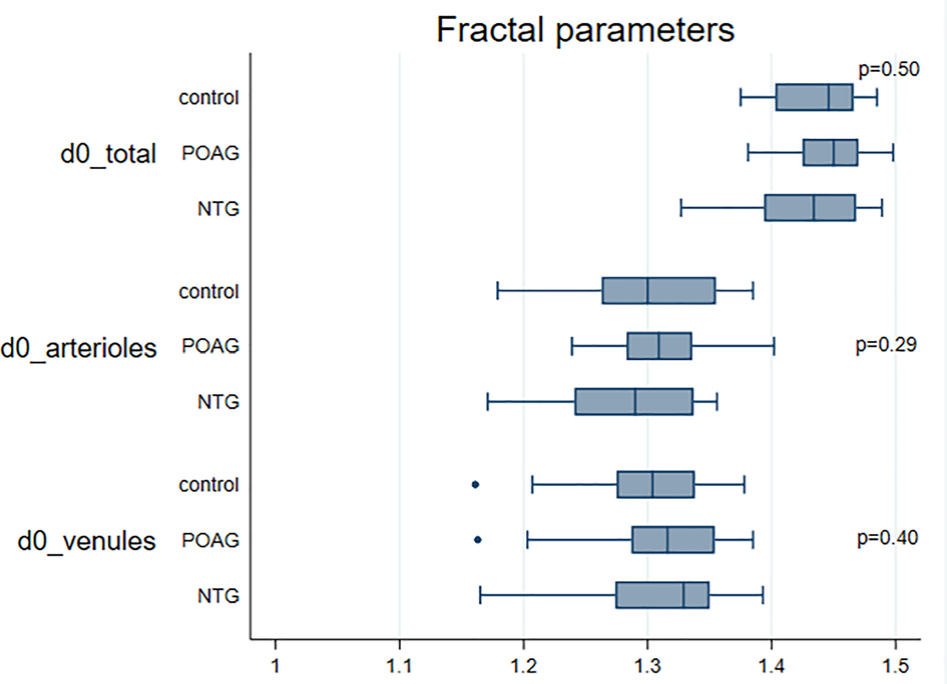
Bar graph of venular tortuosity values in the NTG, POAG and control groups. Data are expressed as median (interquartile range, 25–75th percentiles). *d0* fractal dimension, *NTG* normal tension glaucoma, *POAG* primary open angle glaucoma.

Median values for CRAE, CRVE, AVR obtained with VAMPIRE were 130.6 µm (122.8; 137.0), 172.1 µm (160.0; 188.3), and 0.76 (0.72; 0.81) in NTG patients (Figs. 2, 3, 4, 5). No significant differences were observed in retinal vessel morphological parameters across study groups.

In NTG and POAG groups, no significant relation was found between CRAE, CRVE, AVR, tortuosity and fractal parameters with RNFL thickness or MD (Table 3).

**Table 3:** Univariable associations between artery/veins characteristics or fractals parameters and retinal nerve fiber layer (RNFL) or mean deviation (MD), by group.

		POAG		NTG	
		RNFL	MD	RNFL	MD
Dependent variables					
CRAE	Regression coefficient	0.3056	− 0.2537	− 0.0015	1.0609
	95% CI	[− 0.2488; 0.6360]	[− 1.1863; 0.6790]	[− 0.8991; 0.8960]	[− 0.8796; 3.0014]
	P value	0.07	0.58	0.99	0.27
CRVE	Regression coefficient	0.2371	− 0.1415	0.3055174	1.527
	95% CI	[− 0.1275; 0.6017]	[− 1.1362; 0.8533]	[− 0.6332; 1.2443]	[− 0.4696; 3.5245]
	P value	0.19	0.77	0.13	0.13
AVR	Regression coefficient	0.0007	− 0.0008	− 0.0012	− 0.0006
	95% CI	[− 0.0013; 0.0028]	[− 0.0062; 0.0046]	[− 0.0033; 0.0009]	[− 0.0055; 0.0042]
	P value	0.45	0.77	0.25	0.79
Arterial tortuosity	Regression coefficient	0.000001	0.000002	0.0000006	0.000003
	95% CI	[0.000001; 0.000004]	[− 0.000005; 0.000009]	[− 0.0000024; 0.0000036]	[− 0.000004; 0.000009]
	P value	0.25	0.62	0.68	0.41
Venular tortuosity	Regression coefficient	0.0000009	0.000001	0.0000002	0.0000006
	95% CI	[− 0.0000002; − 0.000002]	[− 0.000002; 0.0000042]	[− 0.0000008; 0.000001]	[− 0.0000017; 0.0000029]
	P value	0.09	0.38	0.69	0.60
Fractals parameters					
d0 total	Regression coefficient	0.000003	0.001518	0.0011	0.0009
	95% CI	[− 0.0011; 0.0010]	[− 0.0011; 0.0041]	[− 0.0005; 0.0027]	[− 0.0028; 0.0047]
	P value	0.99	0.24	0.17	0.61
d0 arterioles	Regression coefficient	0.0002	0.0006	0.0017	0.0026
	95% CI	[− 0.0012; 0.0017]	[− 0.0031; 0.0044]	[− 0.0004; 0.0038]	[− 0.0024; 0.0075]
	P value	0.72	0.72	0.11	0.290
d0 veinules	Regression coefficient	0.0004	0.0028	0.0015472	0.0009
	95% CI	[− 0.0015; 0.0022]	[− .0020; 0.0076]	[− 0.0006; 0.0036]	[− 0.0041; 0.0058]
	P value	0.69	0.23	0.14	0.72

*AVR* arteriole-to-venule ratio, *CI* 95% confidence interval, *d0* fractal dimension, *CRAE* central retinal arterial equivalent, *CRVE* central retinal venular equivalent, *NTG* normal tension glaucoma, *POAG* primary open angle glaucoma.

## Discussion

Using a matched nested case–control study design, no significant association was found between retinal vessel parameters estimated with VAMPIRE software and NTG.

When comparing NTG patients with controls, our results are consistent with a retrospective Indian study (*n* = 88 subjects including 28 NTG, 30 POAG and 30 controls) which reported no significant difference in retinal vessel diameters of affected quadrants by glaucoma according to the VF compared with that of unaffected quadrants in the same eye of age- and severity-matched NTG or POAG with hemifield damage^27^. In this latter study, vessels calibers were measured using Image J software at a distance of 1.72 mm of the center of the OD. In contrast, in previous studies using semi-automatic software conducted in Asian populations, NTG patients (n = 60) was associated with reduced arterial (109.8 ± 12 vs 120 ± 11.3 µm) and venular (158.5 ± 17.6 vs 176.8 ± 21.1 µm) retinal vascular diameters compared to controls (n = 45, matched for age and sex)^28^. In the Singapore Malay Eye study (n = 3019 subjects including 74 NTG patients)^29^, logistic regression models adjusted for sex, age, smoking status, BMI, serum glucose, systolic blood pressure and hypertension status found that only narrower CRVE was associated with NTG. A Korean study^30^ found that both CRAE and CRVE were reduced in unilateral NTG-affected eyes and unaffected NTG-fellow eyes as compared with controls. However, in this latter study, exclusion of patients or controls with systemic vascular diseases and systemic medications may represent a significant bias.

We also found no significant association between CRAE and CRVE which VAMPIRE measures of arterial and venous retinal vessels with RNFL or VF indices in the two glaucoma groups, which is consistent with previous studies in NTG^27,30^ or POAG^27^. This data is however in contrast with another study which found a significant correlation between RNFL thickness and CRAE in NTG patients (*n* = 60 previously untreated NTG)^28^.

A difficulty inherent to comparing results with previously reported ones is the difference in choices, e.g. retinal measurements to be used, algorithms to compute them, patient cohort characteristics and study design, among others. Within these limitations, a review of relevant work reveals the following. Firstly, the prevalence of NTG in Asians is known to be higher than in Caucasians^31^. Whether the severity of glaucoma, associated systemic and ocular vascular risk factors and genetic backgrounds are different in Caucasians and Asians is not known. Possible differences may explain discrepant associations between the retinal vessel phenotype and NTG. Secondly, some studies were conducted before glaucoma treatment^28,32^. The effect of hypotensive eye drops used by patients with glaucoma on retinal vessel diameter remains controversial. Dervenis et al. reported that the use of ophthalmic medications was associated with increased values of CRAE^33^. Conversely, Wong et al. found that antiglaucoma medications, in particular, topical beta-blockers, were associated with narrower arteriolar and venular diameters in individuals with and without arterial hypertension^34^. Thirdly, our study sample mainly consisted of patients with mild NTG (52.2% had MD < − 6 dB) and retinal vessel diameter may vary depending on glaucoma severity. As summarized in Table 4, visual field loss severity, non-available in some studies, varies with MD between − 4.7 and − 11 dB and RNFL thicknesses between 68 and 89.6 microns.

**Table 4:** Primary studies reporting on retinal vessel parameters for normal tension glaucoma (NTG).

Studies	Our study	Chang et al.^28^	Amerasinghe et al.^29^	Shin et al.^30^	Rao et al.^27^
NTG population	Caucasian, 23 NTG, hospital-based population, 63 years (56; 74)*	Asian, 60 NTG, hospital-based, 53.7 years (± 9)**	Asian, population-based, 74 NTG, mean age of total population, 57.6 years (± 10.6)**	Asian, 37 unilateral NTG, hospital-based, 55.8 years (± 10.7)**	Indian, 28 NTG, 54 years (± 11.4)**
Control group	23 age, systemic hypertension, diabetes, and refraction-matched subjects, hospital-based population, 65 years (55; 74)*	45 age- and sex-matched Asian control subjects, hospital-based, 50.5 years (± 7.2)**	2945 Asian, population-based, mean age of total population, 57.6 years (± 10.6)**	40 age- and sex-matched healthy controls, hospital-based, 57.0 years (± 9.1)**	30 age-matched controls, 54 years (± 4.3)**
Data from the NTG population					
Medical treatment (%)	86.96%	Previously untreated patients	NA	NA	NA
Previous surgical treatment (%)	0%	Previously untreated patients	NA	NA	NA
RNFL (µm)	73 (60; 78)*	89.6 (± 12.9)**	NA	88.6 (± 12.9)	68 (± 48.06)**
MD (dB)	-5.1 (-9.6; -1.6)*	NA	NA	− 4.7 (± 4.3)	-11 (± 4.9)**
CRAE (µm)	130.6 (122.8; 137.0)*	109.8 (± 12.0)**	136.81 (± 13.23)**	154.6 (± 15.8)**	STA = 71 (± 16.8)** ITA = 66 (± 18.1)**
CRVE (µm)	172.1 (160.0; 188.3)*	158.5 (± 17.6)**	209.49 (± 20.41)**	200.8 (± 22.8)**	STV = 108 (± 24.09) ** ITV = 115 (± 31.9)**
AVR	0.76 (0.72; 0.81)*	0.70 (± 0.09)**	NA	0.77 (± 0.06)**	NA

Data are expressed as median (interquartile range (25–75th percentiles))* and mean (standard deviation)**

*AVR* arteriole-to-venule ratio, *CRAE* central retinal artery equivalent, *CRVE* central retinal vein equivalent, *ITA* inferotemporal artery diameter, *ITV* inferotemporal vein diameter, *MD* mean deviation (dB: decibels), *NA* not available, *RNFL* retinal nerve fiber layer, *STA* superotemporal artery diameter, *STV* superotemporal vein diameter, *TRT* treatment.

When comparing NTG with POAG patients, our study was consistent with two other studies which did not show any difference in the mean arteriolar and venular diameters between NTG and POAG^29,35^. Lee et al. reported smaller CRAE but comparable CRVE estimates in NTG relative to POAG eyes^32^.

In POAG, narrowing of arterial and venous diameter has been previously reported in Caucasians^20^ and Asians^29^. Using the same Vampire software (version 3.0.3)^20^ as in the present study, we reported that POAG was associated with a lower values of fractal dimension than controls but no difference for arterial and venous tortuosity. In the Singapore Malay Eye study, POAG was associated with decreased arterial and venous tortuosity and lower FD^36^. A prospective study showed that narrower CRAE was associated with a higher risk of occurrence of POAG, supported the concept that early vascular changes are involved in the pathogenesis of glaucoma^37^. The conflicting results between our previous study in POAG^20^ and the present study may be due to different retinographs and lower resolution of images being used in the latter study whereas age and severity of glaucoma (MD, RNFL thickness) of POAG patients was similar. One should acknowledge that two patients with POAG benefitted from filtering surgery. However, the effect on IOP reduction after deep sclerectomy on CRAE is tiny (minus 6 microns, median)^38^. POAG population significantly differed from the NTG population by a more frequent use of carbonic anhydrase inhibitors (CAI). Since CAI medication have not been reported to have a significant effect on retinal vessel diameters, 4/12/2023 11:58:00 AM this difference in using CAI drops between groups may not have influenced retinal vessel diameters.

Finally, the vascular dysregulation associated with NTG is based on data from the retrobulbar vessels^8,9^, the anterior part of the ONH^7,41–43^, the retina^11,12,44,45^ and the choroid^10^. These studies of different vascular ocular beds with different modes of regulations (myogenic, autonomic innervation) and different modes of explorations (Laser Doppler, Laser speckle flowgraphy, OCT-angiography, OCT-Doppler, fluorescein angiography, dynamic retinal vessel analysis, measurements of retinal vessels) suggest a vascular risk factor for NTG. Other risk factors were reported in the literature, such as low nocturnal blood pressure^46,47^, reduced ocular perfusion pressure^48–51^, vasospasm^52,53^, and migraine.^54,55^ The absence of retinal vascular changes in Caucasian patients does not exclude vascular dysregulation since VAMPIRE measurements focus on the retinal vascular geometry only. These measurements do not enable one to explore vascular reactivity.

The strengths of our study include the high resolution of fundus images and study groups matched by age, refraction, systemic hypertension, diabetes and severity of glaucoma, all parameters which can have an impact on the retinal vasculature^56^. One trained grader (ASL) evaluated all the images (certified in the VAMPIRE training module).

Some limitations of this study should be discussed. First, our study might be underpowered due to the relatively limited sample size. Second, the observational design of the study does not allow to exclude unmeasured confounding factors. Third, medical history was self-reported and we did not use standardized criteria for cardiovascular risk factors, with the potential for misclassification. Fourth, our study was hospital-based: control and cases eyes were retrieved from the database of patients from the same department and a potential Berkson bias could not be excluded^57^.

In conclusion, we did not find significant differences between morphological parameters of the retinal microvasculature (arterial and venous vessels diameters, tortuosity, FD) between NTG, POAG and controls. No relation was found between retinal vascular parameters and MD or RNFL in NTG and POAG groups. These results underline that phenotype of the retinal vessel architecture and diameters using semi-automatic software from fundus images does not reveal an impact of the potential dysregulation of NTG.

## Data Availability

The datasets used and/or analysed during the current study available from the corresponding author on reasonable request.
